# Characteristics of Retinal Reflectance Changes Induced by Transcorneal Electrical Stimulation in Cat Eyes

**DOI:** 10.1371/journal.pone.0092186

**Published:** 2014-03-20

**Authors:** Takeshi Morimoto, Hiroyuki Kanda, Tomomitsu Miyoshi, Yoko Hirohara, Toshifumi Mihashi, Yoshiyuki Kitaguchi, Kohji Nishida, Takashi Fujikado

**Affiliations:** 1 Department of Applied Visual Science, Osaka University Graduate School of Medicine, Suita, Osaka, Japan; 2 Department of Integrative Physiology, Osaka University Graduate School of Medicine, Suita, Osaka, Japan; 3 Research Institute, Topcon Corporation, Itabashi-ku, Tokyo, Japan; 4 Innovative Research Initiatives, Tokyo Institute of Technology, Yokohama, Kanagawa, Japan; 5 Department of Ophthalmology, Osaka University Graduate School of Medicine, Suita, Osaka, Japan; Charité University Medicine Berlin, Germany

## Abstract

Transcorneal electrical stimulation (TES) activates retinal neurons leading to visual sensations. How the retinal cells are activated by TES has not been definitively determined. Investigating the reflectance changes of the retina is an established technique and has been used to determine the mechanism of retinal activation. The purpose of this study was to evaluate the reflectance changes elicited by TES in cat eyes. Eight eyes of Eight cats were studied under general anesthesia. Biphasic electrical pulses were delivered transcornealy. The fundus images observed with near-infrared light (800–880 nm) were recorded every 25 ms for 26 s. To improve the signal-to-noise ratio, the images of 10 consecutive recordings were averaged. Two-dimensional topographic maps of the reflective changes were constructed by subtracting images before from those after the TES. The effects of different stimulus parameters, e.g., current intensity, pulse duration, frequency, and stimulus duration, on the reflective changes were studied. Our results showed that after TES, the reflective changes appeared on the retinal vessels and optic disc. The intensity of reflectance changes increased as the current intensity, pulse duration, and stimulation duration increased (*P*<0.05 for all). The maximum intensity of the reflective change was obtained when the stimulus frequency was 20 Hz. The time course of the reflectance changes was also altered by the stimulation parameters. The response started earlier and returned to the baseline later with higher current intensities, longer pulse durations, but the time of the peak of the response was not changed. These results showed that the reflective changes were due to the activation of retinal neurons by TES and might involve the vascular changes induced by an activation of the retinal neurons.

## Introduction

Transcorneal electrical stimulation (TES) activates retinal neurons and evokes electrical potential changes in the visual cortex. These changes lead to visual sensations in humans [1]–[3]. TES is used clinically for the treatment of optic neuropathy and retinal diseases [4]–[7] and for the determination of residual retinal function in patients with advanced retinitis pigmentosa (RP) [8]–[11]. Although electrophysiological studies have been performed to try to determine how TES activates retinal neurons [12], [13], it has not been determined definitively because it is difficult to record electrically evoked retinal responses because of the artifacts induced by the electrical stimuli [14].

*In situ* optical imaging of the intrinsic signals is a well-established method of studying brain physiology and mapping the functional architecture of the cerebral cortex [15]–[17]. The intrinsic signals are represented by the optical reflectance changes associated with the activity of neural tissues [15]–[17]. The reflectance changes of neural tissues have been attributed to the physiological oximetric changes [16], [17], blood flow changes [16], [18], and light-scattering changes [19].

Optical imaging of the intrinsic signals of the retina has also been extensively studied [20]–[31], and it has become an established technique to investigate how the retina is activated by electrical currents [22], [24], [27], [30]. However, the details of how the electrical stimuli affect the reflectance changes have not been determined.

Thus, the purpose of this study was to evaluate the retinal reflectance changes elicited by different stimulation parameters of the TES in cat eyes. We shall show that the reflectance changes appeared on the blood vessels and the optic disc, and the intensity of the reflectance changes was dependent on the parameters of the electrical stimuli. These reflective changes were due to the activation of retinal neurons by TES and might involve the vascular changes induced by an activation of the retinal neurons.

## Materials and Methods

### Ethics Statement

All experiments were performed in accordance with the ARVO Statement for the Use of Animals in Ophthalmic and Vison Research, and all procedures were approved by the Animal Research Committee of Osaka University graduate school of medicine.

All efforts were made to minimize suffering.

### Animals and Preparation

Eight healthy adult cats between 7 months and 1-year-of-age of both sexes and weighing 2.5–3.0 kg were studied. These cats were raised in a breeding colony in the Institute of Laboratory Animals, Osaka University, Graduate School of Medicine. The cats were initially anesthetized with an intramuscular injection of ketamine HCl (25 mg/kg) followed by an intraperitoneal injection of atropine sulfate (0.1 mg/kg). The anesthesia and paralysis were maintained with a continuous intravenous infusion of sodium pentobarbital (1.0 mg/kg/hr), pancuronium bromide (0.2 mg/kg/hr), glucose (0.1 g/kg/hr) in Ringer’s solution at a rate of 5 ml/hr. The cats were artificially ventilated with a mixture of N_2_O/O_2_ (1∶1), and the end-tidal CO_2_ concentration was kept at 3.5 to 5.0% by altering the frequency and tidal volume of the ventilation. The intratracheal pressure and electrocardiogram were also monitored. The body temperature was maintained at 38°C with a heating pad.

Only the left eyes of the cats were studied due to equipment and space limitations. The pupil was dilated with a solution of 0.5% tropicamide and 0.5% phenylephrine hydrochloride, and 1% atropine. A closed ring shaped eyebar made of stainless steel was used to stimulate the eye. The ring (diameter, 16.5 mm ) of the eyebar was sutured to the limbus of cornea, and the end of the bar of the ring was attached to the stereotaxic headholder to minimize eye movements. To protect the corneal surface, a hard contact lens (polymethylmethacrylate; base curve, 8.50 mm; diameter, 13.5 mm; power, +1.5 diopters) was placed on the cornea. The corneas were kept moist with drops of a 0.9% NaCl solution during the experiment.

### Optical Imaging of Reflectance Changes of Retina

The ocular fundus was viewed with a fundus camera (TRC-50LX, Topcon, Tokyo, Japan) equipped with a digital CCD camera (C8484, Hamamatsu Photonics, Hamamatsu, Japan) [22], [31]. The resolution of the camera was 1280×1024 pixels, but the use of the binning mode of the camera to obtain maximum light sensitivity reduced the resolution to 320×256 pixels (12-bit gray scale). A 12-bit digitizer was used, and 4096 grayscale values (GSVs) were obtained for each pixel. The exposure time of the CCD imaging was 20 ms.

A halogen lamp was used to illuminate the posterior fundus, and a band-pass filter was inserted into the optical path to limit the wavelength of the fundus monitoring light to 800–880 nm [22], [31]. The power of the monitor light was 250 nW, which was much lower than the safe exposure limit of the American National Standards Institute.

Fundus images were obtained every 25 ms for 26 s. The images were recorded 2 s before, 4 s during, and 20 s after the electrical stimulation. To improve the signal-to-noise ratio, ten images of ten consecutive measurements were averaged [22], [31]. The interval between sessions was 1 min. A two-dimensional image of the reflectance changes was obtained by subtracting the images recorded before the stimulation from those recorded after the stimulation. A stabilized power supply (PS-150UE-DC, Hayashi Watch Works, Tateyama, Japan) was used to reduce background fluctuations of the illuminating light. All experiments were performed in a dark room after 30 min of dark-adaptation.

### Transcorneal Electrical Stimulation

The left eyes were stimulated transcornealy by the sutured stainless eyebar shaped electrode at the sclerocorneal limbus as one of the electrodes. The return electrode was placed under the skin of the head. The TES consisted of rectangular anodic first (cornea positive) biphasic current pulses obtained from an electrical stimulation system (Stimulator: SEN-7203, Nihon Kohden, Tokyo, Japan; Isolator: BSI-950, Dagan, Minneapolis, MN, USA). To examine the relationship between the stimulus parameters and the intensity of the reflectance changes, the stimulus parameters were changed: current intensities of 0.1,0.5, 1.0, and 2.0 mA of 5 ms/phase at 20 Hz at 20 pulses; pulse durations of 0.5,1.0, 2.0, 3.0, 5.0, and 10.0 ms/phase with constant current intensity at 20 Hz at 20 pulses; frequencies of 5, 10, 20, 30, and 50 Hz at constant current intensity of 5 ms/phase at 20 pulses; and stimulation duration of 0.5, 1, and 4 sec of 5 ms/phase at 50 Hz.

### Light Stimulation of Retina

The eye was stimulated with a 4° vertical bar at 8 Hz for 4 s. The bar was presented 6° temporal to the area centralis. The light power was 30 nW.

### Electrophysiological Recordings from Optic Chiasma

The tips of a pair of stainless steel electrodes was placed in the optic chiasma (OX) stereotaxically to record the electrical potential changes evoked by electrical stimulation of the retina. This was done to record the potential changes in the axons of the retinal ganglion cells elicited by TES. The electrode was inserted from the cortical surface at 13–14 mm anterior to the ear bard and 1–2 mm ipsilateral to the midline. The depth of the electrode tip was 23–26 mm from the cortical surface. Light-evoked responses were recorded from each electrode to be certain that the electrodes were placed in the OX.

To record the electrically evoked potentials (EEPs) by TES, the signal was amplified 10,000 times and bandpass filtered between 300 Hz and 5 kHz with an AC amplifier (Model 1800; Microelectrode AC amplifier; A-M Systems, Inc., Carlsborg, WA) and a signal conditioner (LPF-202A; Warner Instruments, Hamden, CT). Amplified EEPs were fed to a signal processor (Power 1401; Cambridge Electronic Design, Cambridge, UK) and were analyzed with a sampling frequency of 50 kHz offline. Signals were also monitored on an oscilloscope and an audio speaker in real time.

The amplitudes, latencies, and implicit times of the EEPs evoked by TES was measured. The amplitude was measured between the first negative through (N1) to the first positive peak (P1). The P1 latencies were also measured.

### Data Analyses

To evaluate the intensity of the reflectance changes, the GSV of each spot of the retina within the fundus image was averaged. The averaged GSV of each spot after the onset of electrical stimulation was subtracted from that before the stimulation to obtain the change in the reflectance of the image. Each recording trial consisted of 300 video frames collected at 30 frames/s for a total recording time of 26 s. The GSVs of 15 video frames collected in 0.5 second were averaged for individual data points to determine the time course of the stimulation-induced reflectance changes. The data were used to plot the time courses of the reflectance changes.

The amplitude was calculated as poststimulus GSV/0.5-second pre-stimulus GSVs pixel by pixel as the relative reflectance changes. The relative reflectance changes were normalized for comparison among the cats, because the magnitude of reflectance changes varied for each cat.

We also analyzed the time course of reflectance changes to determine the latency as the start of the rise of the wave of the reflectance changes, the implicit time as the time to the peak of reflectance changes, the time when the reflectance change returned to the baseline.

### Statistical Analyses

Data were analyzed with the JMP (ver. 9.0; SAS Institute Inc., NC) program. The data are expressed as the means ± standard error of the means. Regression analyses between the stimulus parameters and the intensity and latency of the reflectance changes or the amplitude and latency of the EEP at the OX was evaluated by JMP. Statistical significance was set at *P*<0.05.

## Results

### Characteristics of Retinal Reflectance Changes after TES

**Figure 1:**
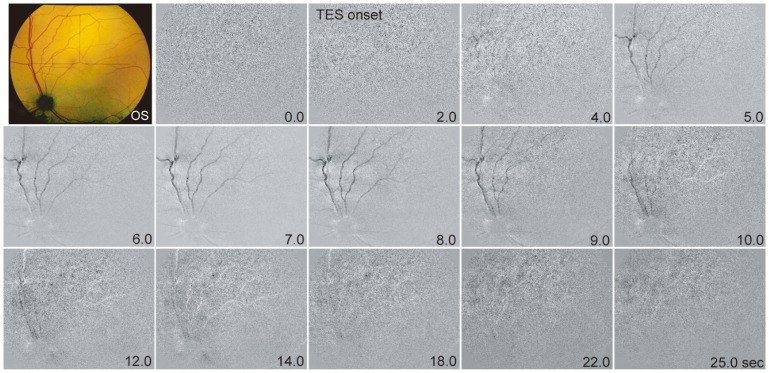
Images of the fundus of the eye of a cat showing reflectance changes in response to transcorneal electrical stimulation (TES). The reflectance changes appeared on the optic disc (OD) and over the retinal blood vessels after the TES. The reflectance changes began about 2.0 s after the onset of stimulation (4.0 s after the onset of recording), and the intensity of the reflectance change increased for 7.0 s after the onset of the recording. It then gradually decreased for 12.0 s. The top left is a fundus photograph of the left eye.

Two-dimensional maps of the reflectance changes after TES are shown in Figure 1. The reflectance changes appeared at the optic disc (OD) and retinal blood vessels about 2.0 s after the onset of stimulation (4.0 s after the onset of recording). The intensity of the reflectance changes continued to increase for 7.0 s and then gradually decreased for 12.0 s after the onset of the recordings.

**Figure 2:**
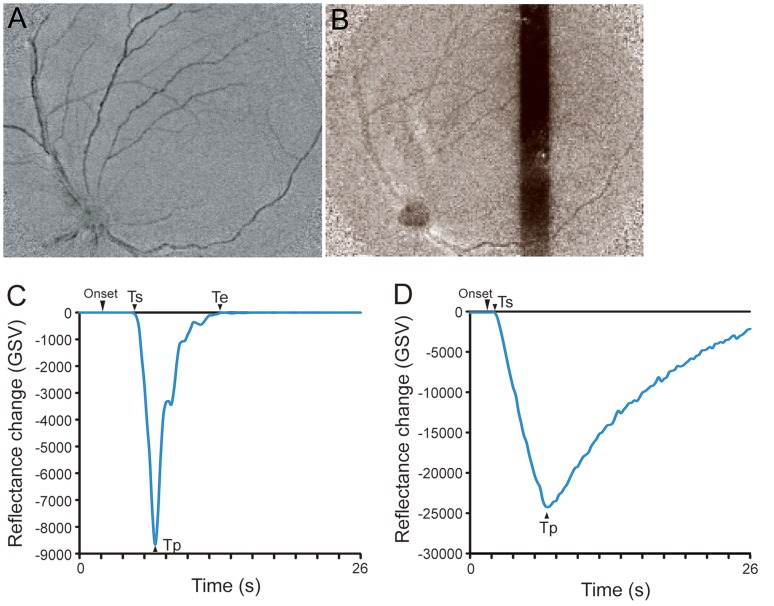
Images of reflectance changes elicited by TES (A) and by photic stimulation (B). The reflectance changes on the OD and over the retinal blood vessels decreased after TES (A), while reflectance changes evoked by light stimulation appeared at the light-stimulated retinal area, the retinal vessels, and OD (B). Plot of the time course of the reflectance changes evoked by TES (C) and light stimulation (D). The latency of the light stimulated retina (D) was shorter than that after TES (C), but the implicit time of the response was same. The time to return to the baseline in the light stimulated retina (D) was significantly later than that after TES (C).

Images of the reflectance changes after TES and light stimulation are compared in Figures 2A and 2B. The time course of the reflectance changes evoked by TES and light stimulation is shown in Figures 2C and 2D. There were some differences of images and time courses in the reflectance changes between TES and light stimulation. The GSV of the reflectance changes evoked by TES began to decrease about 5.0 s after the onset of recording and continued to decrease for 7.0 s (Fig. 2C). The GSV returned to the baseline at about 13.0 s (Fig. 2C).

The reflectance changes evoked by light stimulation appeared at the light-stimulated retinal area, the retinal vessels, and optic disc (OD) (Fig. 2B). The time course of the GSV of the reflectance changes evoked by light stimulation are plotted in Figure 2D. The GSV of the reflectance changes decreased just after the onset of light stimulation, and the latency of the reflectance changes (Ts) after the light stimulation was shorter than that after TES, but the implicit time of the peak of the reflectance changes (Tp) was the same. However, the time to return to the baseline (Te) in the light stimulated retina was significantly longer than that after TES (Figs. 2C, 2D).

### Effect of Current Intensity of TES on Reflectance Changes

**Figure 3:**
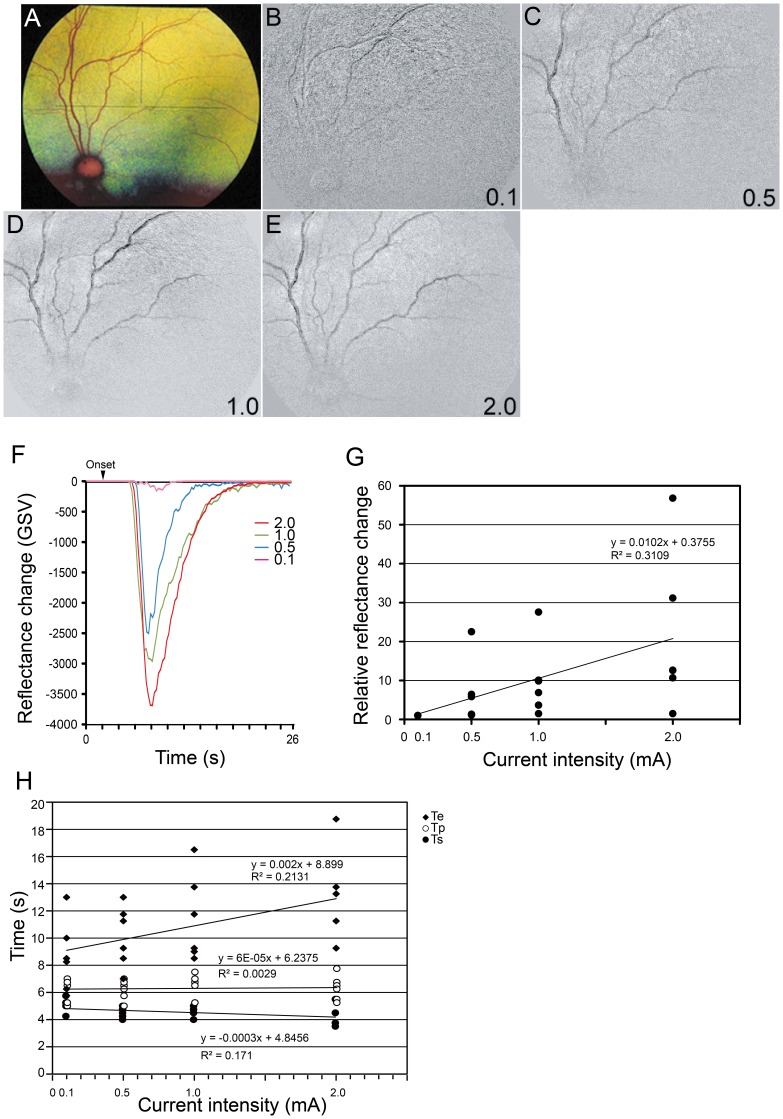
Effect of current intensity of TES on reflectance changes. Fundus photograph (A) and images of reflectance changes elicited by different current intensities (B–E). The GSVs of the reflectance changes (dark signal) decreased as the current intensity increased (F). The relative reflectance changes increase as the current intensities increase (G). The increase in the maximum relative reflectance changes as the current intensities increase was almost linear with stimulus currents up to 2.0 mA (G). There was a significant positive correlation between relative reflectance changes and current intensities (r^2^ = 0.311, *P* = 5×10^−4^). A plot of the latency and current intensity is graphed in H. The latency decreases and time to return to baseline increases as the current intensity increases. The changes in the latency is significantly correlated with the current intensities (r^2^ = 0.1333, *P* = 0.045). The reflectance changes are also significantly correlated with the current intensities (r^2^ = 0.213, *P* = 002) (H).

The effect of the strength of the electric current on the reflectance changes was determined for currents from 0 to 2.0 mA. The stimulus duration was 5.0 ms/phase, frequency was 20 Hz, and stimulation duration was 1.0 s. The GSV of the reflectance changes increased with an increase of electric current (Figs. 3A to 3F). The maximum relative reflectance changes increased almost linearly with an increase in the stimulus current up to 2.0 mA (Fig. 3G). Simple regression analysis showed that there was a significant positive correlation between relative reflectance changes and current intensities (r^2^ = 0.311; *P = *0.005; Fig.3G).

Three measurements of the time course of the reflectance changes are plotted in Figure 3H. It can be seen that the latency of the reflectance changes (Ts) and the time to return to the baseline (Te) were depended on the current intensity. There were significant correlations between these latencies and current intensities (Ts, r^2^ = 0.133, *P = *0.045; Te, r^2^ = 0.213; *P* = 0.023; Fig. 3H).

### Effect of Pulse Duration on Reflectance Changes

**Figure 4:**
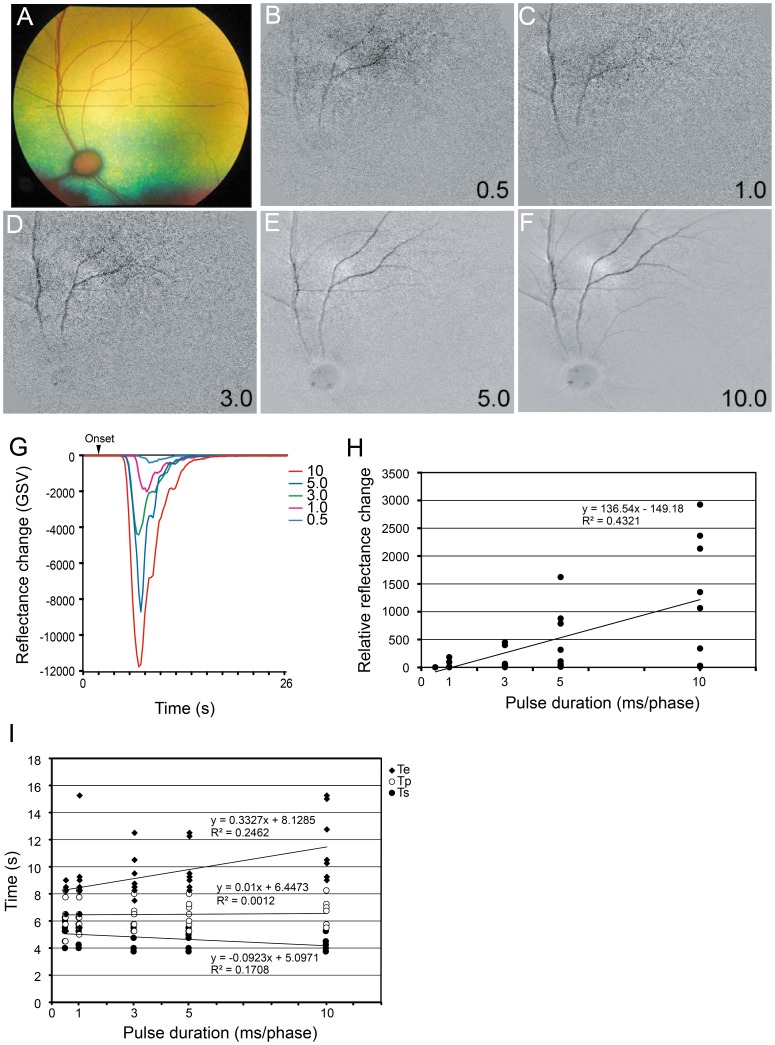
Effect of stimulus pulse duration on reflectance changes. Fundus photograph (A) and images of reflectance changes elicited by different pulse durations (B–F). The GSV of the reflectance changes (dark signal) decreases as the pulse duration increases (G). The relative reflectance changes increases as the pulse durations increase (H). The relative reflectance changes increases almost linearly with an increase of the pulse duration from 0.5 to 10.0 ms/phase (G). There is a significant positive correlation between the relative reflectance changes and pulse durations (r^2^ = 0.432, *P*<1×10^−4^). Plot of the relationship between the latency and pulse duration (H). The latency decreases as the pulse duration increases, but the time to return to the baseline increases as the pulse duration increases. The latency is significantly correlated with the pulse duration (r^2^ = 0.171, *P* = 0.009), and the time to return to baseline was significantly correlated with the pulse durations (r^2^ = 0.246, *P* = 0.001) (l).

The effect of pulse duration on the reflectance changes was determined for pulse durations of 0.5 to 10.0 ms/phase. These experiments were done with a pulse frequency of 20 Hz; current intensity of 0.1 to 0.5 mA depending on the response; and stimulus duration time of 1.0 s. Our findings showed that the reflectance changes decreased with increasing pulse durations up to 10.0 ms/phase (Figs. 4A to 4G). The increase in the amplitudes of the relative reflectance changes depended on the pulse duration (Fig. 4H). Simple regression analysis showed that there was a significant positive correlation between the relative intensities of the reflectance changes and pulse durations (r^2^ = 0.432; *P*<0.0001).

The latency of a response (Ts) and time to the return to the baseline (Te) depended on the pulse duration (Figure 4I). The implicit times did not change significantly with pulse duration. There were significant correlations between the latency and time to return to baseline of the reflectance changes and pulse durations (Ts, r^2^ = 0.171, *P* = 0.009; Te, r^2^ = 0.246, *P* = 0.001; Fig. 4I).

### Effect of Stimulation Duration on Reflectance Changes

**Figure 5:**
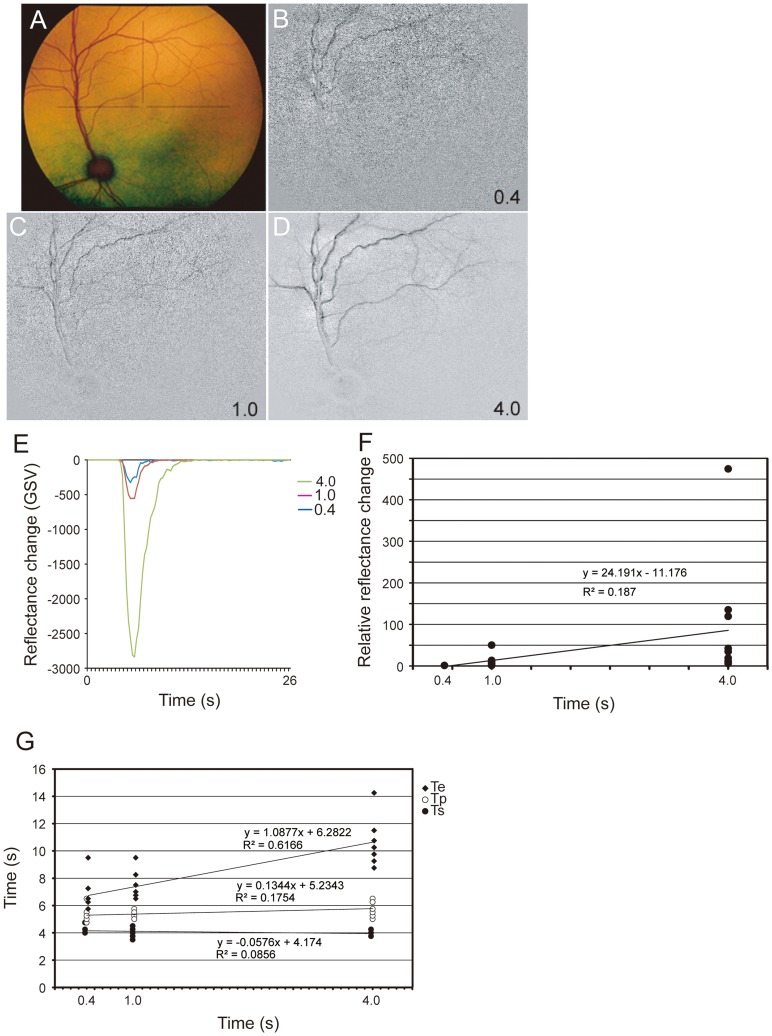
Effect of stimulation duration on reflectance changes. Fundus photograph (A) and images of the reflectance changes elicited by different stimulation durations (B–D). The GSV of reflectance changes (dark signal) decreases as the stimulation duration increases (E). Relative reflectance changes increases as the stimulation duration increases (F). There was an almost linear increase in the maximum relative reflectance changes and the stimulation duration from 0.4 to 4.0 s (F). There was a significant positive correlation between relative intensities of reflectance changes and the stimulation duration (r^2^ = 0.187, *P* = 0.017). The relationship between latency and the stimulation time (G). The time to return to baseline increases as the stimulation duration increases. There was a significant correlation between the time to return to baseline and the stimulation duration (r^2^ = 0.086, *P*<1×10^−4^) (G).

The effect of stimulation duration on the reflectance changes was determined for stimulus durations of 0.4, 1.0, and 4.0 s. For these experiments, the pulse frequency was 50 Hz, current intensity was 0.1 to 0.5 mA depending on the response, and the pulse duration was 5 ms/phase. The relationship between the reflectance changes and the stimulation time showed that the reflectance changes also decreased almost linearly with the stimulation durations up to 4.0 s (Figs. 5A to 5E). The amplitudes of the relative reflectance changes increased and was depended on the stimulation duration (Fig. 5F). Simple regression analysis showed that there was a significant positive correlation between the relative intensities of the reflectance changes and stimulation duration *(*r^2^ = 0.187, *P*<0.017). The time course of the reflectance changes are plotted in Figure 5G. The latency (Ts) and time to baseline (Te) increased as the stimulation duration increased. There was a significant positive correlation between the times (Ts, Te) and the stimulation duration (Ts, Te: r^2^ = 0.086, *P*<0.0001).

### Effect of Stimulus Frequency on Reflectance Changes

**Figure 6:**
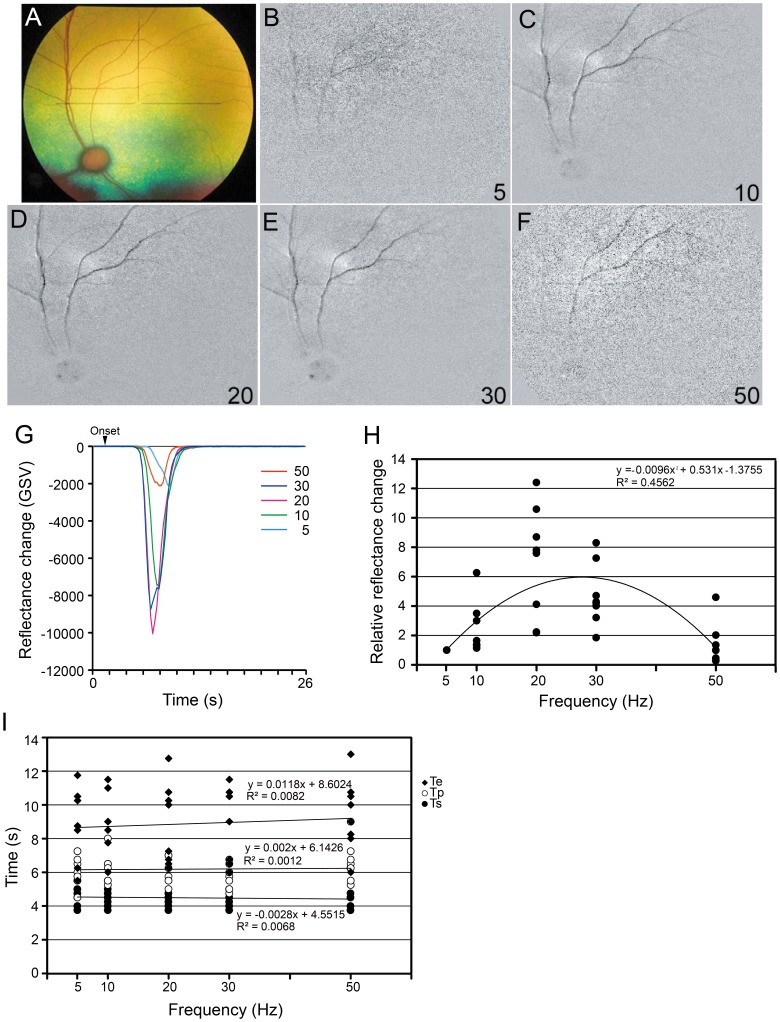
Effect of stimulus frequency on reflectance changes. Fundus photograph (A) and images of reflectance changes elicited by different stimulus frequencies (B–F). The GSV of the reflectance changes (dark signal) depended on the frequency (G), with the relative reflectance changes depended on the stimulus frequency (H). The maximum relative reflectance changes was best fit to the stimulus frequency by a non-linear curve (quadratic term; *P* = 0.001). The relationship between the latency and the frequencies (I). There was no significant correlation between latencies and stimulus frequencies.

The effect of the stimulus frequency ranging from 5 to 50 Hz on the reflectance changes was determined with a current intensity of 0.1 to 0.5 mA depending on the response, pulse duration of 5 ms/phase, and pulse number of 20. The relationship between reflectance changes and the stimulus frequency showed that the GSV of the reflectance changes was also dependent on the stimulus frequency (Figs. 6A to 6G). The amplitudes of relative reflectance changes also changed depending on the frequency (Fig. 6H). Non-linear polynomial regression analysis showed that these data were best fitted by a non-linear equation (quadratic term; *P* = 0.001; Fig. 6H). The latencies of Ts, Tp, and Te are plotted in Figure 6I. There was no significant correlation between all of these measures and the stimulus frequencies.

### Electrophysiological Recordings from Optic Chiasm after TES

**Figure 7:**
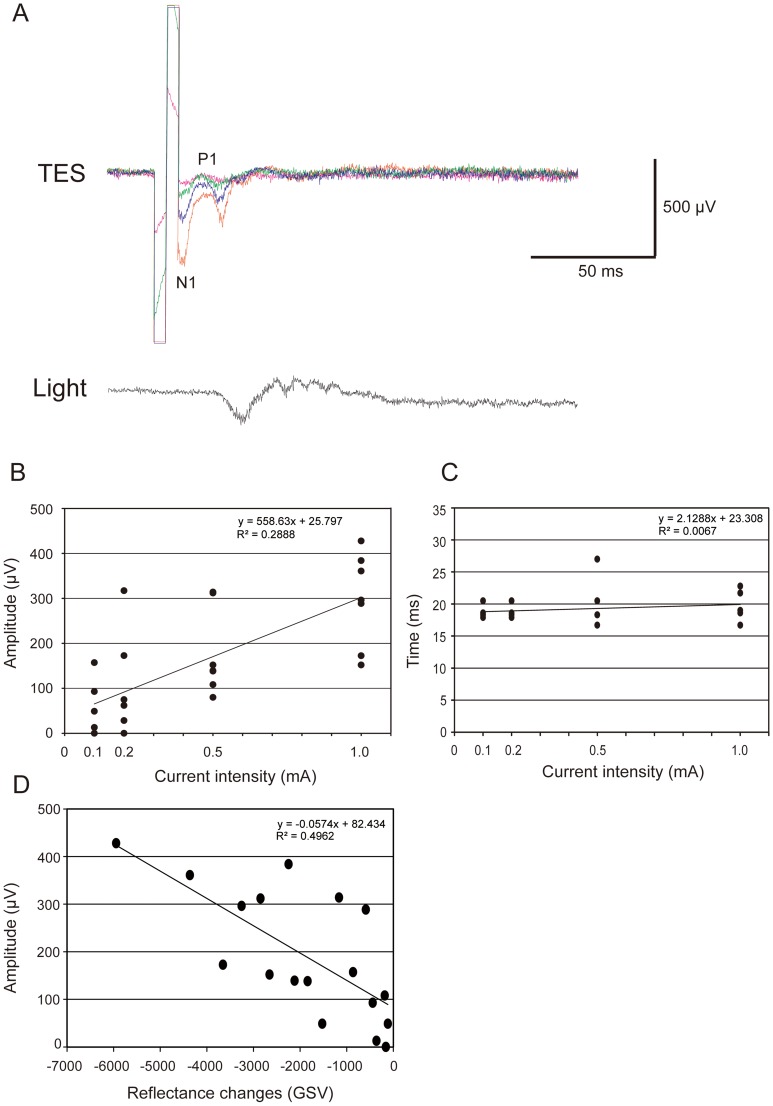
Electrophysiological recordings from the optic chiasm (OX) after TES. The electrically evoked potentials (EEPs, upper) and the light evoked potentials (VEPs, lower) are shown. Graph showing the relationship between the EEP amplitude and electric current (B) and the relationship between the peak latency (P1) and electric current (C). B. There was a significant positive correlation between amplitudes of EEPs and current intensities (*P* = 0.0007, r^2^ = 0.2888). C. The relationship between the peak latency (P1) and electric current. There was no significant correlation between latencies and current intensity. D. EEP amplitude was correlated with the GSV of reflectance change in the retina. There was a significant positive correlation between intensities of reflectance changes and EEP amplitudes (r^2^ = 0.496, *P* = 0.001).

We examined the relationship between the EEP amplitude recorded in the OX and the intensity of the stimulus currents. The EEP amplitude increased linearly with an increase of stimulus current (Figs. 7A, 7B), and linear regression analysis showed that there was a significant positive correlation between amplitudes of EEPs and the current intensities (r^2^ = 0.289; *P* = 0.001). However, there was no significant correlation between the P1 latencies and the current intensities (Fig. 7C).

These results indicate that TES with the parameters used can activate retinal ganglion cells (RGCs) directly or indirectly and the degree of activation was significantly correlated with the intensity of electrical current. In addition, the EEP amplitudes were significantly correlated with the GSV of the reflectance changes of the retina *(P* = 0.0011; r^2^ = 0.4962; Fig. 7D). This suggests that the reflectance changes represented the neuronal changes in the retina, i.e., the excitation of the RGCs.

## Discussion

Our results showed that there were specific reflectance changes of the retina in response to the TES. The GSVs of the reflectance changes were significantly increased with higher current intensities, longer pulse durations, and longer stimulation durations. These findings indicate that the increase in the GSVs of the reflectance changes was due to the increase of the electrical flux. In addition, there was a frequency specificity with the maximum signals obtained with a stimulus frequency of 20 Hz, if the electrical flux was same at each frequency. Our findings are similar to those obtained from monkey eyes [24], [28].

The relationship between the time course and stimulus parameters on the RCs has not been determined. We have determined the relationship between the time course and stimulus parameters on the RCs. The time course of the responses was also altered by the parameters of the TES. The latency of response (Ts) was shorter and the time to return to the baseline (Te) was longer with higher current intensities and longer pulse durations, but the time of the peak of the response (Tp) was not changed (Figs. 3H, 4I). When the current intensity and the pulse duration were the same, only the time to return to baseline (Te) was longer with longer stimulation durations (Fig. 5G).

An increase in the electrical flux by stronger currents, longer pulse durations, and longer stimulation durations should increase the number of retinal neurons activated by TES. Therefore, the latency of the reflectance changes are shorter and the return to the baseline longer. Thus, the time course of the response might be related to the electrical flux. On the other hand, the peak implicit time of the response (Tp) was not changed by the different parameters of the TES. This indicates that the reflectance changes occurred in response to a certain process, such as a neurological change to a vascular change.

### What do Intrinsic Reflectance Changes Represent?

The reflectance changes evoked by TES appeared over the retinal vessels and optic disc, and these were different from those elicited by photic stimulation (Fig. 2s). Mihashi et al reported that the inhibition of the action potentials of retinal ganglion cells (RGCs) by tetrodotoxin (TTX) abolished the reflectance changes elicited by TES or by OX stimulation, but TTX did not induce significant changes in the retinal intrinsic signals elicited by photic stimulation [31]. These results suggest that the reflectance changes after either TES or OX stimulation were caused by the activation of the RGCs.

Although there was a significant correlation between the activity of RGCs and the reflectance changes, this does not directly indicate that they are due to the activation of the RGCs, because the latency of the response was much longer than that of action potentials of RGCs. The RCs represent the responses associated with hemodynamic changes in either the blood volume and flow or the oxygenated state of hemoglobin that is caused by the activity of the RGCs [32]–[35].

The existence of a fundamental relationship between neural activity, blood flow, and metabolism, called a neurovascular coupling, was suggested by Roy and Sherrington [32]. A neurovascular coupling in the optic nerve and retina was postulated by Riva et al [33]. The blood flow and local partial pressure of oxygen in the optic nerve have been shown to be modulated by intermittent light stimuli, and both were coupled to the activity of RGCs [34], [35]. These findings indicate that the blood flow and metabolism in the retina and optic nerve can be modulated by local neural activity. Moreover TES has been shown to increase the chorioretinal blood flow in normal subjects with minimal effects on the systemic blood circulation and the intraocular pressure [36]. The increased retinal blood flow elicited by photic stimulation measured by laser Doppler flowmetry is similar to that of the reflectance changes induced by the same stimuli [28]. Thus, the reflectance changes investigated in this study represented the hemodynamic responses of the retina and optic nerve to the increased retinal neural activity, which are secondary to the activation of the neural activity of the retina. We suggest that the RCs represent the response of neurovascular coupling and TES might stimulate the neurovascular coupling in the retina and optic nerve.

In conclusion, the intensities of the reflectance changes were dependent on the stimulus parameters of TES. The reflectance changes represent changes of the retinal vessels and optic disc, and these results indicate that TES influenced neurovascular coupling, i.e., RGCs and retinal hemodynamics. More experiments are necessary to determine the retinal neurovascular coupling, i.e., the relationship between the activated RGCs and the retinal hemodynamics. However, we conclude that this imaging technique might be a method to investigate the neurovascular coupling.
